# Correlation Between the Cost and Safety of Corneal Graft Types

**DOI:** 10.7759/cureus.55435

**Published:** 2024-03-03

**Authors:** Panagiotis S Kousiouris, Maria Kantzanou, Maria Dantsiou, Amalia Drosopoulou, Konstantinos Rallis, Dimitrios Papakonstantinou, Marilita M Moschos

**Affiliations:** 1 First Department of Ophthalmology, National and Kapodistrian University of Athens, Medical School, Athens General Hospital “Georgios Gennimatas”, Athens, GRC; 2 Department of Hygiene, Epidemiology and Medical Statistics, National and Kapodistrian University of Athens, Medical School, Athens, GRC; 3 Department of Purchasing, Athens General Hospital “Georgios Gennimatas”, Athens, GRC; 4 First Department of Ophthalmology, Athens General Hospital “Georgios Gennimatas”, Athens, GRC; 5 State Department of Ophthalmology, Athens General Hospital “Georgios Gennimatas”, Athens, GRC; 6 First Department of Ophthalmology, National and Kapodistrian University of Athens, Medical School, Athens, GRC; 7 First Department of Ophthalmology, National and Kapodistrian University of Athens, Athens, GRC

**Keywords:** keratoconus (kc), fuchs endothelial corneal dystrophy, public health policy, cornea pathology, cornea transplantation

## Abstract

Background

Corneal diseases are the fourth most common cause of blindness worldwide. In the majority of these diseases, vision reduction is reversible and can be restored to a large extent by replacing the cornea through specific surgery and, in particular, transplantation. In Greece, due to a lack of organized eye banks as well as donors, the grafts intended for corneal transplantation usually come from eye banks abroad. This study focuses on the dynamics of cost versus value in the decision-making process for the procurement of corneal grafts, ultimately investigating the safety that the procured grafts provide to patients.

Methodology

A total of 267 patients with severe vision problems who underwent 301 corneal and amniotic membrane transplants from years 2020 to 2023 at the Transplant Unit of the Athens General Hospital "Georgios Gennimatas” were included in this study. All patients who were deemed appropriate to undergo corneal transplant operations, the diagnosis that led to the specific surgery, and other relevant data were recorded and evaluated.

Results

There was no significant difference in the ratio between males and females (51.3% male and 48.7% female). The mean age of the patients was 66.5 years (SD = 13.7 years). Graft problems were faced by 13.9% of the patients, with the amniotic membrane by 1.5% (in the total number of surgical operations) and both eyes by 4.5% of patients. The majority of the patients had undergone only one surgery (88.8%). Reoperation was needed in 14% of the cases, and 7.6% of the cases were surgeries that occurred due to graft rejection or non-functioning grafts from surgeries performed at another hospital or clinic. In the majority of surgeries (60.8%), a Descemet’s stripping automated endothelial keratoplasty graft was used. The mean cost was 3,167 euro (SD = 960.3 euro). Furthermore, in 35.8% of the surgeries, the graft was preserved with amphotericin.

Conclusions

The present study draws useful conclusions about the effectiveness of surgical interventions through the correlation of cost and safety of the grafts that are approved and finally used in corneal transplants, as well as the submission of proposals to improve the procedures and lead to patient benefits.

## Introduction

It is estimated that more than 280 million people worldwide are suffering from low vision or blindness in both eyes [1]. Corneal diseases are the fourth most common cause of blindness worldwide [2,3]. In the majority of these diseases, vision reduction is reversible and can be restored to a large extent by replacing the cornea through a specific surgical operation, transplantation [3-5]. Many corneal diseases cause vision loss and they include various types [5]. Damage to the cornea that alters the shape of the cornea (such as keratoconus, which is a condition where the cornea is cone-shaped rather than dome-shaped) or its transparency through opacities (e.g., in corneal stroma dystrophies, in corneal edema due to dystrophy Fuchs endothelium or in bullous keratopathy after cataract, glaucoma or vitrectomy operations, in scars due to infections or injuries, etc.) results in reduced vision, which in some cases lead to blindness. In these cases, as well as in cases of severe thinning of the cornea or inflammation that cannot be treated with drugs, the only solution to restore the integrity of the bulb and improve vision is a corneal transplant [5-8]. Corneal transplantation, which is also known as keratoplasty, can involve the replacement of the entire cornea (transparent) or a part of it (partial thickness), depending on the condition [6].

In Greece, due to the lack of organized eye banks and the lack of donors, the grafts intended for corneal transplantation usually come from foreign eye banks [9-13]. These banks have very strict operating regulations, providing the grafts only after detailed and complete quality control [14-16].

This retrospective study which is the first in the literature that brings to light the correlation of cost and safety of corneal graft types aims to focus on the dynamics of price versus value in the decision-making process for the procurement of human tissue grafts to meet corneal transplant needs, ultimately studying the safety of the procured grafts for patients undergoing the relevant procedures (transplants).

## Materials and methods

Methodology

A total of 267 patients with severe vision problems who underwent 301 corneal and amniotic membrane transplant operations from 2020 to 2023 (recorded cases up to July 2023) at the Transplant Unit of the Athens General Hospital “Georgios Gennimatas” were included in this study. Data regarding all cases who were deemed appropriate to undergo corneal transplant operations, the diagnosis that led to the specific surgery, as well as the control of the restoration of their vision after a reasonable period were recorded and evaluated. Among them, 30 patients who underwent corneal transplantation in 2023 who could be contacted were asked about their postoperative status. All patients who received a donor graft were included in our study and none was excluded.

Statistical analysis

Quantitative variables were expressed as mean (SD), while qualitative variables were expressed as absolute and relative frequencies. For the comparison of proportions, Fisher’s exact tests were used. Student’s t-tests and analysis of variance were computed to compare mean values [17-21]. Bonferroni correction was used to control for type I errors [22]. All reported p-values are two-tailed. Statistical significance was set at p-values <0.05. Data analyses were conducted using SPSS statistical software version 26.0 (IBM Corp., Armonk, NY, USA).

## Results

**Figure 1:**
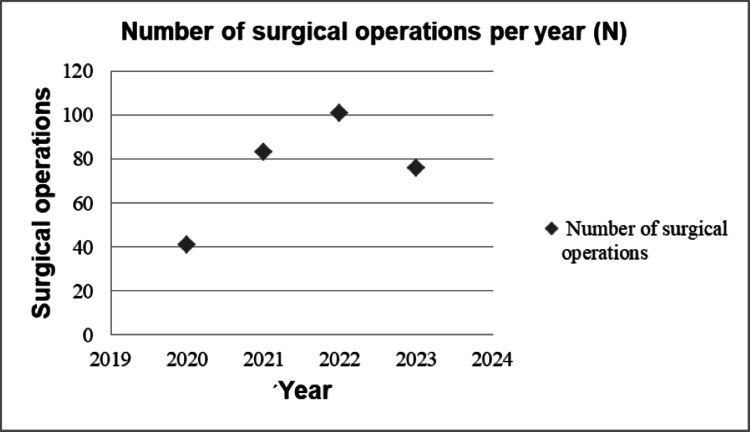
Number of surgical operations per year.

Data from 301 surgeries of 267 patients performed between 2020 and 2023 were recorded. Table 1 and Figure 1 show the surgeries performed from 2020 to 2023 (up to July 2023).

**Table 1:** Distribution of interventions per year.

Year of surgery	Number of surgeries (Ν)	N (%)
2020	41	13.6
2021	83	27.6
2022	101	33.6
2023	76	25.2

The considerably smaller number of surgical operations during the year 2020 is related to the appearance of the COVID-19 pandemic [23-25] (since March 2020), where the fear of an unknown disease, the difficulties relating to the protection from this disease, the Greek Government’s measures of restriction (prohibition of mass movement and gatherings (lockdowns)) affected the attendance at hospitals (people were afraid of being at risk of another disease, besides the one they were already facing), while the administrations of the hospitals and doctors decreased the number of surgeries.

**Figure 2:**
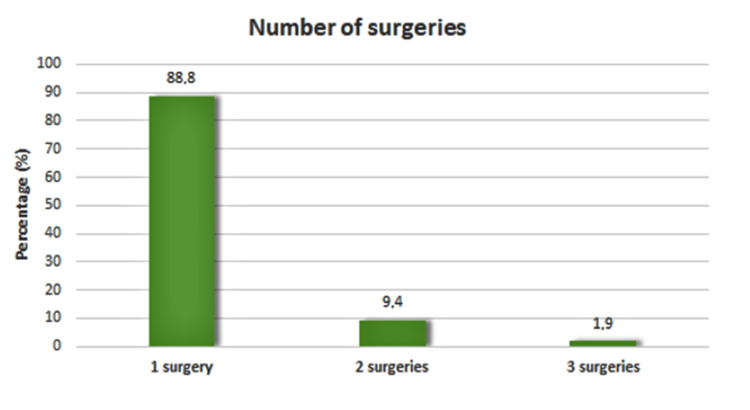
Number of surgeries performed per patient.

**Figure 3:**
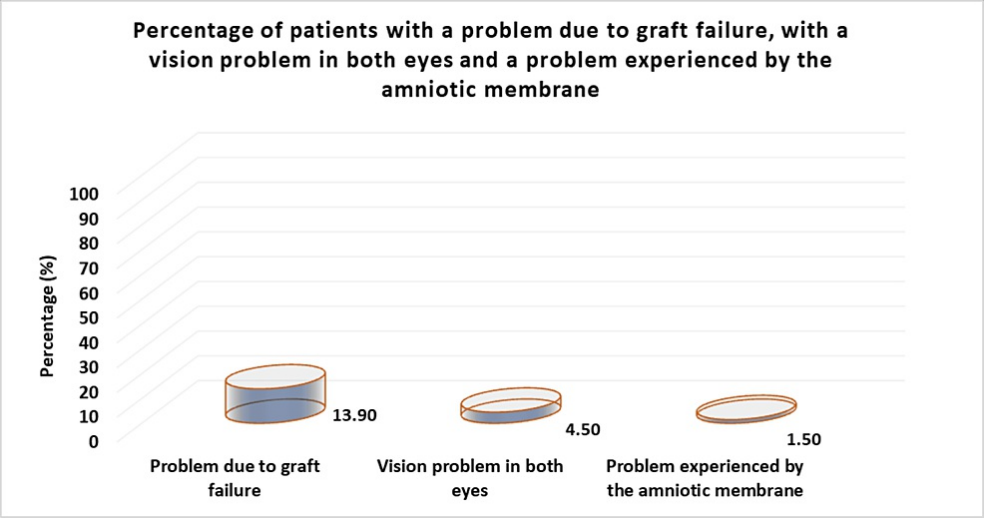
Percentage of patients with a problem due to graft failure, with a vision problem in both eyes, and a problem experienced in the amniotic membrane.

Data related to the transplants that the patients in our study group underwent are shown in Table 2. There was no significant difference in the ratio between males and females (51.3% male and 48.7% female). The mean age of the patients was 66.5 years (SD = 13.7 years). The majority of the patients (88.8%) had undergone only one surgery (Figure 2). Graft problems were faced by 13.9% of the patients, problems with the amniotic membrane were faced by 1.5% of the patients (of the total number of surgical operations), and problems in both eyes were faced by 4.5% of the patients (Figure 3).

**Table 2:** Data related to the transplants that the patients underwent.

Total number of patients who underwent transplants between 2020 and 2023 (Ν = 267)	Ν (%)
Gender	
Male	137 (51.3)
Female	130 (48.7)
Age, mean (SD)	66.5 (13.7)
Problems with graft	37 (13.9)
Problems in both eyes	12 (4.5)
Problems with amniotic membrane	4 (1.5)
Number of surgeries needed	
1	237 (88.8)
2	25 (9.4)
3	5 (1.9)

Types and major characteristics of the surgical operations that the patients underwent are presented in Table 3. Reoperation was needed in 14% of the cases, while 7.6% of the cases were surgeries that occurred due to graft rejection [26,27] or a non-functioning graft from a surgery performed at another hospital or clinic. In the majority of the surgeries (60.8%), a Descemet’s stripping automated endothelial keratoplasty (DSAEK) graft was used. The mean cost was 3,167 euro (SD = 960.3 euro). Furthermore, in 35.8% of the surgeries, the graft was preserved with amphotericin. The use of amphotericin in the graft maintenance fluid was evaluated based on the international literature, as having a significant effect against fungal infections [28,29]. The doctors of the Transplant Ophthalmology Unit of the Athens General Hospital “Georgios Gennimatas” request grafts preserved in liquid Optisol GS containing amphotericin since 2022. These specific grafts are considered to be safer for the patients; however, their costs are higher.

**Table 3:** Type and major characteristics of the surgical operations that the patients underwent. ^1^: not referred to in PK or amniotic membrane surgeries. DALK = deep anterior lamellar keratoplasty; DMEK = Descemet’s membrane endothelial keratoplasty; DSAEK = Descemet’s stripping automated endothelial keratoplasty; PK = penetrating keratoplasty

Total number of surgeries recorded between 2020 and 2023 (Ν = 301)	Ν (%)
Reoperation needed	42 (14.0)
Surgery due to graft rejection or a non-functioning graft from a surgery performed at another hospital	23 (7.6)
Eye	
Right	155 (52.4)
Left	141 (47.6)
Type of surgery	
Amniotic membrane	38 (12.6)
DALK	1 (0.3)
DMEK	16 (5.3)
DMEK precut	1 (0.3)
DSAEK	183 (60.8)
PK	61 (20.3)
PK + Amniotic membrane	1 (0.3)
Amniotic membrane	
Amniotic membrane insertion	4 (11.1)
Amniotic membrane transplantation	10 (27.8)
Amniotic membrane suturing	13 (36.1)
Placement of amniotic membrane	9 (25.0)
Cost, mean (SD)	3,167.0 (960.3)
Amphotericin^1^	72 (35.8)

The correlation of cost with the major characteristics of the transplants that the patients underwent is presented in Table 4. The cost was found to differ significantly among different types of surgery, with lower costs seen in surgeries for amniotic membrane compared to surgeries for Descemet’s membrane endothelial keratoplasty (DMEK) (p < 0.001), surgeries for DSAEK (p < 0.001), and surgeries for penetrating keratoplasty (PK) (p < 0.001). Moreover, the cost of PK surgeries was significantly lower compared to DMEK/DMEK precut (p < 0.001) and DSAEK (p < 0.001). Within surgeries for amniotic membranes, significant differences were found in the specific type, with transplantations having significantly lower costs than those for insertion (p = 0.012). Moreover, the cost was significantly greater when there was a problem in both eyes and when there was not a problem with the amniotic membrane.

**Table 4:** Correlation of cost with major characteristics of the transplants that the patients underwent. ^+^: Student’s t-test; ^++^: analysis of variance; ^1^: not referred in PK or amniotic membrane surgeries. DALK = deep anterior lamellar keratoplasty; DMEK = Descemet’s membrane endothelial keratoplasty; DSAEK = Descemet’s stripping automated endothelial keratoplasty; PK = penetrating keratoplasty

Characteristics of the transplants	Cost		P-value
	Mean	SD	
Reoperation needed			
No	3,200.5	892.1	0.135^+^
Yes	2,961.7	1298.6	
Surgery due to graft rejection or a non-functioning graft from surgery performed at another hospital			
No	3,148.2	983.5	0.238^+^
Yes	3,394.3	583.8	
Eye			
Right	3,145.2	960.6	0.273^+^
Left	3,264.1	892.0	
Type of surgery			
Amniotic membrane	948.2	199.2	<0.001^++^
DALK	2,700.0	-	
DMEK/DMEK precut	3,822.4	198.9	
DSAEK	3,724.3	195.1	
PK	2,687.0	145.2	
PK + Amniotic membrane	3,630.0	-	
Amniotic membrane			
Amniotic membrane insertion	1,200.0	480.0	0.019^++^
Amniotic membrane transplantation	834.0	205.5	
Amniotic membrane suturing	958.7	3.9	
Placement of amniotic membrane	946.7	36.4	
Amphotericin^1^			
No	3,641.2	165.2	<0.001^+^
Yes	3,882.2	191.1	
Problems with graft			
No	3,129.3	968.9	0.155^+^
Yes	3,331.7	912.3	
Problems in both eyes			
No	3,119.3	985.1	0.004^+^
Yes	3,692.4	309.2	
Problems with amniotic membrane			
No	3,204.3	923.8	<0.001^+^
Yes	1,807.5	1,321.9	

The percentage of cases with graft problems was similar in all types of surgery, as presented in Table 5. On the contrary, the percentage of cases with problems in both eyes and with amniotic membranes differed significantly among all types of surgery (p = 0.018 and p = 0.002, respectively). More specifically, a higher percentage of having problems in both eyes was reported in DMEK and DSAEK surgeries. In cases where surgery involved the amniotic membrane, the percentage of cases with the amniotic membrane was higher.

**Table 5:** Problems reported by the type of surgery. ^1^: Fisher’s exact test. DALK = deep anterior lamellar keratoplasty; DMEK = Descemet’s membrane endothelial keratoplasty; DSAEK = Descemet’s stripping automated endothelial keratoplasty; PK = penetrating keratoplasty

Type of surgery	Problems with graft		Problems in both eyes		Problems with the amniotic membrane	
	No	Yes	No	Yes	No	Yes
	N (%)	N (%)	N (%)	N (%)	N (%)	N (%)
Amniotic membrane	32 (84.2)	6 (15.8)	38 (100.0)	0 (0.0)	32 (84.2)	6 (15.8)
DALK	1 (100.0)	0 (0.0)	1 (100.0)	0 (0.0)	1 (100.0)	0 (0.0)
DMEK	15 (93.8)	1 (6.3)	13 (81.3)	3 (18.8)	16 (100.0)	0 (0.0)
DMEK precut	1 (100.0)	0 (0.0)	1 (100.0)	0 (0.0)	1 (100.0)	0 (0.0)
DSAEK	143 (78.1)	40 (21.9)	162 (88.5)	21 (11.5)	181 (98.9)	2 (1.1)
PK	52 (85.2)	9 (14.8)	60 (98.4)	1 (1.6)	61 (100.0)	0 (0.0)
PK + Amniotic membrane	0 (0.0)	1 (100.0)	1 (100.0)	0 (0.0)	1 (100.0)	0 (0.0)
P^1^	0.284		0.018		0.002	

## Discussion

Transplantation is the replacement of a pathological cornea with a clean one obtained from a cadaveric donor after its suitability has been checked. In contrast to what applies to most organs in the body and to other solid organs that are transplanted (e.g., heart, liver, kidneys), the cornea has the advantage of being avascular, which implies that it has no arteries and veins and is fed exclusively by its neighboring fluids [9]. This creates a very important advantage in the case of transplantation, as it can be obtained even when the donor is dead. In addition, it significantly reduces the risk of rejecting the transplant, reducing the chances of being recognized as a foreign body and rejected by the recipient’s body [16].

This study included 267 patients who had undergone 301 operations from 2020 to 2023 (and specifically, up to July of this specific year). The overwhelming majority involved corneal grafts (263), while the remaining operations (38) involved amniotic membranes. Of the corneal transplants, the majority involved DSAEK and PK grafts (specifically, 60.8% were DSAEK and 20.3% were PK).

Vision problems in both eyes were experienced by 12 (4.5%) patients, problems with the grafts (non-functional grafts or rejected grafts) were experienced by 37 (13.9%) patients, with 7.6% concerning non-successful operations performed in other hospitals or clinics and, finally, problems with the amniotic membranes were experienced by four patients (of the 38 operations that involved amniotic membranes). In 35.8% of procedures (excluding PKs and amniotic membranes), all of them from 2022 onward, a graft preserved in liquid Optisol GS containing amphotericin was used. The supply of grafts preserved in liquid Optisol GS containing amphotericin was based on the requirement set by the doctors of the Transplant Ophthalmology Unit of the General Hospital of Athens “Georgios Gennimatas” for the safety of their operated patients’ eyes from fungal infections. However, it should be emphasized that this contributed to the increase in the cost of eye grafts.

The costs of the procedures ranged from 500 euros (amniotic membrane) to 4,100 euros (DSAEK, with the graft preserved in liquid Optisol GS containing amphotericin), with an average cost of 3,167 euros (SD = 960.3 euros ). At this point, it is worth mentioning that the cost of supplying and importing eye grafts from foreign eye transplant banks is currently even higher than the total cost of a corneal transplant operation in a Greek Public Hospital, which is a serious issue that must be taken into consideration and resolved by the Greek Ministry of Health.

Several patients who underwent corneal transplantation in 2023 were contacted (by telephone), after obtaining prior written consent, to get an update on their postoperative status. In these 30 patients, the operation was successful. Some of them already had a significant improvement in their level of vision, while the rest expected an improvement after a reasonable period.

Better knowledge and control of the COVID-19 pandemic, after the first shock of 2020, has contributed to an increase in corneal transplant surgeries.

## Conclusions

This is the first retrospective study in the literature that brings to light the correlation between the cost and safety of corneal graft types. Good work was performed in the Transplantation Unit of the General Hospital of Athens “Georgios Gennimatas”. The restoration of serious vision damage (even total blindness) and the return of every patient to a more active role in society are priceless for many reasons. However, the development and operation of eye transplant banks in Greece would lead to several advantages and better outcomes, such as better (more thorough) control of eye grafts in comparison with the eye grafts supplied from foreign countries, reduced transplant reception times, earlier transplants, reduced waiting time, and, by extension, reduced waiting list of patients. In addition, this will lead to improvement in workload management procedures in operating rooms, with notable positive benefits for transplant recipients and nursing staff. Finally, a significant reduction in the total cost of operations can be achieved, as the cost of importing a corneal graft from a foreign eye transplant bank is currently even higher than the cost of a corneal transplant operation in a Greek public hospital.
